# A combined treatment of Proteinase K and biosynthesized ZnO-NPs for eradication of dairy biofilm of sporeformers

**DOI:** 10.3934/microbiol.2022033

**Published:** 2022-12-19

**Authors:** Ahmed A. Radwan, Osama M. Darwesh, Maha T. Emam, Karima A. Mohamed, Hala M. Abu Shady

**Affiliations:** 1 Genetics and Cytology Dept., National Research Centre (NRC), Cairo, 12622, Egypt; 2 Agricultural Microbiology Dept., National Research Centre (NRC), Cairo, 12622, Egypt; 3 Microbiology Dept., Faculty of Science, Ain-Shams University Cairo, Egypt

**Keywords:** dairy sporeformers, biofilm eradication, proteinase K, biosynthesized ZnO-NPs

## Abstract

Biofilms of sporeformers found in the dairy industry are the major contaminants during processing, as they withstand heat and chemical treatment that are used to control microbes. The present work is aimed to remove these resistant forms of bacterial community (biofilm) present in dairy production lines using ecofriendly agents based on proteinase K (Prot-K) coupled with Zinc oxide nanoparticles (ZnO-NPs). Some metal/metal oxide (Ag, CuO and ZnO) NPs were prepared microbially, and ZnO-NPs were characterized as the most effective ones among them. The produced ZnO-NPs were 15–25 nm in size with spherical shape, and FTIR analysis confirmed the presence of proteins and alkanes surrounding particles as capping agents. Application of Prot-K for eradication (removal) of a model biofilm of mixed sporeformers on food-grade stainless steel resulted in an 83% reduction in the absorbance of crystal violet-stained biofilm. When Prot-K was mixed with the biosynthesized NPs ZnO_G240, the reduction increased to 99.19%. This finding could contribute to an efficient cleaning approach combined with CIP to remove the recalcitrant biofilms in dairy production lines.

## 1. Introduction

Microbes generally tend to form biofilms on all surfaces with sufficient moisture and organic matter supply. Presence of biofilms in the dairy industry raises safety issues, especially when biofilms are located on milk-processing surfaces and pipelines that are unreachable by cleaning agents [1]. Bacteria in biofilms are protected against disinfectants due to the interspecific cooperation and the presence of extracellular polymeric substances (EPS), which enhance their survival and promote the subsequent contamination of dairy products. Indeed, dairy biofilms are composed of specific bacterial species adapted to survive the intrinsic and extrinsic factors (heat, nutrients, pH, salt, etc.) that are associated with milk processing [2]. It is well documented that the bacteria frequently found in the dairy environment, other than the starters, capable of forming biofilms are aerobic sporeformers belonging to genus *Bacillus* and allied genera [3]–[6]. These bacteria are major contaminants in the milk processing industries, as their spores, already existent in raw milk and able to grow at refrigeration temperature, survive pasteurization and subsequent processing, attach to surfaces, form biofilms and consequently become a part of the final product. Their biofilms, especially in the problematic regions (joints, pipe corners, gaskets, etc.), remain after Clean-In-Place (CIP) practices that result in more production of the spoilage enzymes which give rise to off-flavors and structural defects, as well as high numbers of bacteria in the end products, limiting the shelf life and thus leading to huge economic losses [7],[8]. So, in order to remove the formed biofilms of these nonstarter bacteria completely to prevent the regeneration possibility in the subsequent batches, deep cleaning methods are required.

Proteolytic treatment of biofilm is a preferred approach due to proteinaceous contents of the biofilm cells and EPS matrix. Previous studies have been published concerning enzyme degradation of mature biofilms using proteinase K (Prot-K) [9]. It is a very reactive serine protease and stable in a wide range of conditions, including temperature, pH, detergents and buffer salts [10]. As a result, Prot-K is an excellent choice for biofilm disassembly among proteases [11]. Additionally, nanoparticles (NPs) are considered a promising tool for removing bacterial biofilms. Many interactions have been determined between NPs and biofilm, such as electrostatic, hydrophobic and steric, that lead to disruption and prevention of the biofilm growth. These interactions are affected mainly by the size and surface charge of the particles and the structure and composition of EPS matrix [12],[13]. Metal oxide nanoparticles, such as ZnO and CuO, are among the most promising NPs and widely investigated for the treatment of bacterial biofilms [13]. The chemical synthesis of promising NPs is relatively expensive and might result in low biocompatibility and risks to living organisms because of the dangerous compounds that are used. On the other hand, biosynthesis using microorganisms, enzymes or plants has been proposed as possible environmentally sustainable method [13],[14]. The objective of the current study is to assess the susceptibility of dairy biofilms of sporeformers to Prot-K and some biosynthesized metal/metal oxide NPs (Ag, CuO and ZnO) and their combination effects. To achieve this, isolation of the nonstarter dairy biofilm-forming bacteria post-pasteurization (thermoduric and/or sporeformers) was done to form a biofilm model of dairy industries for the study's experiments.

## 2. Materials and methods

### 2.1. Isolation of biofilm-forming bacteria

Five samples (two swab samples, one raw milk and two powdered milk) were collected. Table 1 describes the samples' characteristics and their step(s) of stock solution preparation. Isolation of the biofilm-forming bacteria was carried out by the pour-plate technique on Plate Count Agar (PCA) medium [15], supplemented with 0.2% soluble starch for enhancement of the bacterial spore germination [16]. First, samples of stock solutions were serially diluted 10-fold in sterile distilled water to 10^−5^. Afterward, 0.1 mL of volume from each dilution was poured under an aseptic condition in duplicate onto Petri dishes containing melted PCA medium and allowed to solidify at room temperature. The dishes after agar solidification were incubated for 16 h to 72 h at 37 °C and 55 °C. After incubation, the morphologically different colonies were picked and purified by streaking on new media.

Pure colonies were tested for their ability to form a biofilm on food-grade 316 stainless steel (SS-316) by growing each isolate in 10 mL sterile Tryptic Soy Broth (TSB) containing a coupon (size 10-by-20-mm; grade 316) placed in a Falcon tube (15 mL) and incubated under their optimum growth temperatures. After incubation, the coupons were washed twice by dipping and rinsing using sterile distilled water, transferred to a sterile Falcon tube (50 mL) containing 2 g of glass beads (diameter 5 mm) and 10 mL of 0.1% peptone water and then vortexed for 1 min. Next, the recovered bacterial cells from coupons were diluted and plated on Nutrient Agar (NA). Thereafter, a screening was done for the obtained cultures by Gram staining and microscopic examinations.

**Table 1.** Characteristics and stock solution preparation of the isolation samples.

Sample	Type	Code	Characteristics	Stock preparation
1	Swab	Sw80	Swab samples were taken from two different processing tanks in a dairy manufacturing plant located in El Obour City (Al Qalyubia, Egypt). The first was the retentate tank of the ultrafiltration unit that is downstream of the primary pasteurization (HTST, 80 °C/30 s); however, the second tank was of calcium chloride addition downstream of the extra pasteurization (HTST, 72 °C/30 s). For each sample, three randomly collected swabs were taken as replicates.	Stock solutions of swab samples were prepared by cutting off and transferring the swab cotton-tips to 10 mL of sterile 0.1% peptone water and mixing for 1 min on a vortex mixer.
2		Sw72		
3	Raw milk	RM	A fresh milk sample of cow was obtained from a private farm.	Milk sample (100 mL) was vortexed, and 10 mL aliquot was transferred to a sterile tube and then subjected to HTST pasteurization (75 °C/30 s).
4	Powdered-milk	PMa	Milk powder samples, products of American and Polish companies named NATURE'S FIRST INC and KASKAT LTD, respectively, were purchased from a wholesale outlet, as they were widely used in dairy production at Egyptian companies during the time of the samples' collection.	Ten grams of each powder sample were reconstituted in 90 mL of sterile 0.1% peptone water and agitated for 30 min in an ice bath. Then, individually, 10 mL aliquot was transferred to sterile tubes and subjected to HTST pasteurization (75 °C/30 s).
5		PMp		

### 2.2. 16S rDNA and biochemical identification

The bacterial isolates were identified on the basis of 16S ribosomal RNA gene sequence using two sets of universal primers, 27F (5′-AGAGTTTGATCMTGGCTCAG-3′) and 1492R (5′-TACGGYTACCTTGTTACGACTT-3′), that amplify about 1500 bp of the 16S rDNA region [17]. Genomic DNA was extracted using a GeneJET Genomic DNA Purification kit (Thermo Scientific, USA) according to the manufacturer's instructions. PCR was performed by adding 40 ng of the extracted DNA in 50 µL of PCR reaction solution (1 U of MyTaq™ DNA polymerase (Bioline, Meridian Bioscience Inc., USA), 1x MyTaq buffer contains dNTPs and MgCl_2_, and 10 pmol of each primer). PCR product was purified by QIAquick Gel Extraction Kit (Qiagen, Germany) and sequenced by capillary DNA sequencing systems, Applied Biosystems™ 3730XL (Applied Bio-systems, USA; service provided by GATC Biotech AG, Germany). Obtained sequences were aligned on the blastn program of NCBI (https://blast.ncbi.nlm.nih.gov/Blast.cgi) using the database of 16S ribosomal RNA sequences (Bacteria and Archaea) in rRNA/ITS databases and against the online tool SepsiTest BLAST (http://www.sepsitest-blast.de/en/index.html) for identification to the species level.

Biochemical identification using the analytical profile index (API) method was performed by API 50CH, API 20E strips and CHB/E medium, (bioMerieux, Marcy-l′Etoile, France), following the manufacturer's instructions. Briefly, freshly grown bacterial colonies were taken from each isolate and suspended in saline solution. From that, ampoules of API CHB medium (10 mL) and API 20E (5 mL of 0.85% NaCl) were inoculated using manual pipette to a turbidity equivalent to 2 McFarland. Then, the suspension was applied to API strips and covered with mineral oil in accordance with instructions. The results of color changing after incubation for 48 h were analyzed with API WEB.

### 2.3. Biosynthesis of metal/metal oxide NPs

Green synthesis of metal/metal oxide NPs was carried out using cell free filtrate of 14 thermoalkali actinobacteria species (provided by Dr. Ahmad S. El-Hawary, Faculty of Science, Al-Azhar University) according to methods described by Darwesh & Elshahawy [18] and Darwesh *et al*. [19]. Silver nitrate (AgNO_3_, Sigma-Aldrich, USA) 0.1%, copper sulfate (CuSO_4_.5H_2_O, Merck, Germany) 1% and zinc sulfate (ZnSO_4_.7H_2_O, Sigma-Aldrich, USA) 1% w/v were used as precursors for the biosynthesis. Fresh cultures of the 14 actinobacteria species were prepared by inoculating two disks from actively growing agar culture in 50 mL nutrient broth (NB) with a final pH of 8.5 and incubation at 55 °C and 150 rpm overnight. Then, the cells were harvested and re-suspended in 5 mL PBS. The suspensions were used to inoculate 100 mL complex medium (Glucose, 1%; Yeast extract, 0.5%; Peptone, 0.25%; Casein, 0.25%; MgSO_4_, 0.03%; FeSO_4_, 0.002%; ZnSO_4_, 0.02%; CaCO_3_, 0.1%; KH_2_PO_4_, 0.1%; K_2_HPO_4_, 0.1%) and incubated for 96 h at 55 °C and 200 rpm [20]. After incubation, the grown cultures were filtered by Whatman paper grade 5. Next, equal volumes of AgNO_3_, CuSO_4_ and ZnSO_4_ solutions were mixed with the obtained supernatants for each culture separately. Afterwards, the reaction mixture was incubated in dark conditions at room temperature overnight. Detection and selection of the synthesized NPs were done by visual observation of the color changing and the precipitation at the flask bottom. Further, the NPs were collected by centrifugation at 10, 000 rpm for 15 min, and the pellets were washed 3 times with deionized water and absolute ethanol and dried in a hot air oven at 45 °C. Finally, the powders were re-suspended in deionized water, sonicated and subjected to biofilm eradication (removal) assay.

### 2.4. Biofilm formation assay

The SS-316 coupons were first washed with 0.1% (w/v) SDS, deionized water and 70% ethanol sequentially and then sterilized by autoclaving. Following that, biofilm strains were grown in NB medium at 37 °C to early log phase (3–6 h). The obtained bacterial cells were collected and re-suspended in a sterile saline solution (0.85% NaCl). Then, the suspensions were used either individually or in combination (mixed-species) to inoculate 5 mL of 3% reconstituted skim milk (RSM) with a final concentration of 10^5^–10^6^ CFU/mL in a Falcon tube (15 mL) containing the sterilized coupons placed vertically and incubated-shaken at 150 rpm and 37 °C for 72 h. In order to determine the background of the staining and fouling layer of milk without bacteria, control A (coupon incubated in un-inoculated water) and control B (coupon incubated in un-inoculated skim milk), respectively, were treated the same as in the biofilm formation test.

### 2.5. Crystal Violet (CV) staining assay

Evaluation of the mature biofilm and quantification of the remainder after treatment were accomplished by CV assay [21]. Biofilm masses on the surfaces of the stainless steel coupons were first washed with PBS and fixed with methanol for 15 min. Then, the coupons were transferred to 12-well plates, air dried and stained for 20 min with 1% CV solution. Afterwards, dyes were discarded, and the coupons were rinsed 5 times with distilled water and air-dried again. Subsequently, the coupons were immersed in 5 mL glacial acetic acid (33%) for 10–15 min to de-stain the stained biofilm. Finally, biofilm quantity was detected by transferring the de-stained solution to a disposable cuvette and measuring the absorbance at 590 nm using a spectrophotometer (SHIMADZU, UV-240, Japan).

### 2.6. Biofilm eradication assay

Once incubation of the biofilm strains (mixed-species) with the SS-316 coupons was completed, the culture media were discarded; and the coupons were rinsed thrice with PBS (1X) to remove the non-biofilm cells, then transferred to Falcon tubes (15 mL) containing 5 mL solution of eradication treatment (i. e., Prot-K, biosynthesized NPs or Prot-K + biosynthesized NPs) and incubated for 30 min. The Prot-K was commercial proteinase K of Bioline Co. (Bioline, Meridian Bioscience Inc., USA). The Prot-K treatment alone was performed in 50 mM Tris-Cl (pH 7.8) at 55 °C, whereas the NPs were suspended in water and incubated at room temperature. However, the combination (Prot-K + biosynthesized NPs) was conducted as a one-step procedure using Prot-K buffer and temperature. Each treatment was replicated (3 replicates), and the controls (+ ve and – ve) of removal activity were distilled water without inoculation. After incubation, the coupons were rinsed with PBS and subjected to the staining assay.

### 2.7. Characterization of the effective NPs (ZnO_G240)

The effective NPs in the biofilm eradication assay (ZnO_G240) were subjected to identification by TEM, FTIR and XRD instruments. For examination of size and morphological shape, characterization was done by high-resolution transmission electron microscopy (HRTEM, JEOL 2100, Japan). The sample solution was drop-coated onto the carbon-coated copper TEM grid and loaded after drying into the specimen holder. Then, an HRTEM micrograph was taken, and the size and shape were recorded [22]. In the case of the FTIR instrument, the probable biomolecules involved in capping, reduction and efficient stabilization of the synthesized NPs were recorded using Fourier Transform Infrared Spectroscopy (FTIR, Agilent Cary 630 FTIR spectrometer) in diffuse reflection mode. Sample powder was placed in a micro cup with an inner diameter of 2 mm and loaded into the FTIR spectrometer set at 26 ± 1 °C. Then, the sample was scanned with the infrared light in the range of 400 to 4000 cm^−1^. The resulting spectral data was compared to the reference chart to identify the presented functional groups [23]. The crystal structure of the biosynthesized NPs was characterized using an X-Ray Diffractometer (XRD, Shimadzu XRD-6000). This analysis was done with the nickel-filter and Cu-Kα X-ray target, under the conditions of a 2θ scan range from 10 to 80°, step size of 0.02°, scan rate of 0.5 sec and copper anode source [24].

### 2.8. Toxicity assay of bio-synthesized ZnO_G240

According to Rajabi *et al*. [25] and Saleh *et al*. [26], a brine shrimp toxicity test was performed on the bio-synthesized ZnO_G240 nanoparticle concentrations. 3.3 g of instant ocean sea salt (Aquarium System, Ohio) was dissolved in 100 mL of distilled water, and 0.5 g of the dried cysts of *Artemia salina* (Linnaeus) nauplii was added to the salt solution and incubated at room temperature under continuous aeration and illumination. The larvae (nauplii) hatched within 48 h were distributed by glass capillary in a vial containing 5 mL of sea water. Then, different concentrations (0, 10, 30, 50, 70, 90 µg/mL) of the bio-synthesized ZnO_G240 were prepared in 5 ml of sea water as triplicates, and ten nauplii of *A. salina* were introduced to each concentration and incubated at room temperature for 24 h, followed by 24 h for confirmation. The survival percentage was obtained after counting and recording the number of alive and dead nauplii in each concentration.

### 2.9. Statistical analysis

The collected data from three replicates were statistically analyzed by MINITAB statistical software version 18.1 (Minitab, Inc., PA, USA). One-way analysis of variance (ANOVA) was used to determine the significances through the Tukey test with significance level (*P* value < 0.05).

## 3. Results and discussion

### 3.1. Isolation and identification of the nonstarter dairy biofilm-forming bacteria post-pasteurization

The samples were collected, prepared and cultured based on the study's purpose regarding the ability to form biofilms on stainless steel surfaces in dairy production lines post-pasteurization. Approximately 22 thermoduric or spore-derived colonies were picked randomly and purified for further characterization. Colony description, cell morphology and Gram staining were performed and resulted in obtaining 10 different isolates. According to Gram staining and microscopic observation, the obtained isolates from five samples were Gram positive bacilli, rod-shaped and purple-violet colored. Moreover, all the isolates were mesophilic thermoduric bacteria, and they could grow in a range between 30 and 55 °C and optimally at 37 °C [27]. The isolates were identified by 16S rRNA gene sequences and the biochemical tests of API 50CH and 20E (Table S1). Sequences of the ten isolates were analyzed and deposited at NCBI GeneBank under accession numbers OM857595-OM857604. The results of identification based on 16S rDNA and API biochemical analysis are summarized in Table 2. As expected, all of the isolates were aerobic, spore-forming bacteria and identified as members of *Bacillus* and related species. These findings are consistent with those of Yuan *et al*. [4], Sadiq *et al*. [5], Reginensi *et al*. [7], Zhao *et al*. [28] and Vanderkelen *et al*. [29].

**Table 2.** Identified biofilm-forming strains using 16S rDNA and API biochemical analysis.

Sample	Code	16s rDNA sequence BLASTn			API 50CHB/20E		GenBank deposition	
		Closest species		Identity (%)	Significant Taxa	ID (%)	Strain name	Accession number
Swab after HTST (80 °C/30 s)	Sw80/1	*NCBI*	*Bacillus subtilis* (NR_027552.1)	99.91	*Bacillus subtilis*/ *amyloliquefaciens*	99.9	*Bacillus subtilis* str. Sw80/1	OM857595
			*Bacillus subtilis* (NR_112116.2)	99.83				
		*SensiTest*	*Bacillus subtilis* subsp. *subtilis* (AJ276351)	99.9				
			*Bacillus mojavensis* (AB021191)	99.7				
			*Bacillus subtilis* subsp. *spizizenii* (AF074970)	99.7				
	Sw80/2	*NCBI*	*Brevibacillus brevis* (NR_041524.1)	99.42	*Brevibacillus* non-reactive	99.8	*Brevibacillus brevis* str. Sw80/2	OM857596
			*Brevibacillus formosus* (NR_113801.1)	99.42				
		*SensiTest*	*Brevibacillus brevis* (AB271756)	99.4				
			*Brevibacillus formosus* (AB112712)	99.4				
			*Brevibacillus choshinensis* (AB112713)	98.8				
Swab after HTST (72 °C/30 s)	Sw72/3	*NCBI*	*Bacillus amyloliquefaciens* (NR_116022.1)	99.86	*Bacillus subtilis*/ *amyloliquefaciens*	99.9	*Bacillus amyloliquefaciens* str. Sw72/3	OM857597
			*Bacillus amyloliquefaciens* (NR_112685.1)	99.72				
		*SensiTest*	*Bacillus amyloliquefaciens* (AB255669)	99.7				
			*Bacillus atrophaeus* (AB021181)	99.7				
			*Bacillus vallismortis* (AB021198)	99.6				
	Sw72/4	*NCBI*	*Bacillus subtilis* subsp. *subtilis* (NR_102783.2)	99.88	*Bacillus subtilis*/ *amyloliquefaciens*	99.8	*Bacillus subtilis* subsp. *subtilis* str. Sw72/4	OM857598
			*Bacillus subtilis strain* (NR_027552.1)	99.88				
		*SensiTest*	*Bacillus subtilis* subsp. *subtilis* (AJ276351)	99.9				
			*Bacillus subtilis* subsp. *spizizenii* (AF074970)	99.6				
			*Bacillus mojavensis* (AB021191)	99.6				
	Sw72/5	*NCBI*	*Bacillus coagulans* (NR_041523.1)	99.91	*Bacillus coagulans*	99.9	*Bacillus coagulans* str. Sw72/5	OM857599
			*Bacillus coagulans* (NR_115727.1)	99.72				
		*SensiTest*	*Bacillus coagulans* (AB271752)	99.9				
Raw milk	RM/6	*NCBI*	*Bacillus subtilis* (NR_027552.1)	99.54	*Bacillus subtilis*/ *amyloliquefaciens*	99.6	*Bacillus subtilis* str. RM/6	OM857600
			*Bacillus subtilis* subsp. *subtilis* (NR_102783.2)	99.27				
		*SensiTest*	*Bacillus subtilis* subsp. *subtilis* (AJ276351)	99.5				
			*Bacillus mojavensis* (AB021191)	99.4				
			*Bacillus subtilis* subsp. *spizizenii* (AF074970)	99.3				
	RM/7	*NCBI*	*Bacillus licheniformis* (NR_116023.1)	99.13	*Bacillus licheniformis*	99.0	*Bacillus licheniformis* str. RM/7	OM857601
			*Bacillus licheniformis* (NR_116023.1)	99.13				
		*SensiTest*	*Bacillus licheniformis* (CP000002)	99.1				
			*Bacillus sonorensis* (AF302118)	98.8				
			*Bacillus aerius* (AJ831843)	98.4				
Powdered milk	PMa/8	*NCBI*	*Bacillus sonorensis* (NR_113993.1)	99.90	*Bacillus licheniformis*	-	*Bacillus sonorensis* str. PMa/8	OM857602
			*Bacillus sonorensis* (NR_025130.1)	99.80				
		*SensiTest*	*Bacillus sonorensis* (AF302118)	99.8				
			*Bacillus licheniformis* (CP000002)	99.2				
			*Bacillus aerius* (AJ831843)	98.6				
	PMp/10	*NCBI*	*Bacillus paralicheniformis* (NR_137421.1)	100.0	*Bacillus licheniformis*	99.9	*Bacillus paralicheniformis* str. PMp/10	OM857603
			*Bacillus haynesii* (NR_157609.1)	99.42				
		*SensiTest*	*Bacillus aerophilus* (AJ831844)	99.1				
			*Bacillus sonorensis* (AF302118)	99.0				
			*Bacillus licheniformis* (CP000002)	98.8				
	PMp/11	*NCBI*	*Bacillus subtilis* subsp. *subtilis* (NR_102783.2)	99.91	*Bacillus subtilis*/ *amyloliquefaciens*	91.3	*Bacillus subtilis* subsp. *subtilis* str. PMp/11	OM857604
			*Bacillus subtilis* (NR_027552.1)	99.91				
		*SensiTest*	*Bacillus subtilis* subsp. *subtilis* (AJ276351)	99.9				
			*Bacillus subtilis* subsp. *spizizenii* (AF074970)	99.7				
			*Bacillus mojavensis* (AB021191)	99.7				

### 3.2. Determination of biofilm formation capability

**Figure 1.**
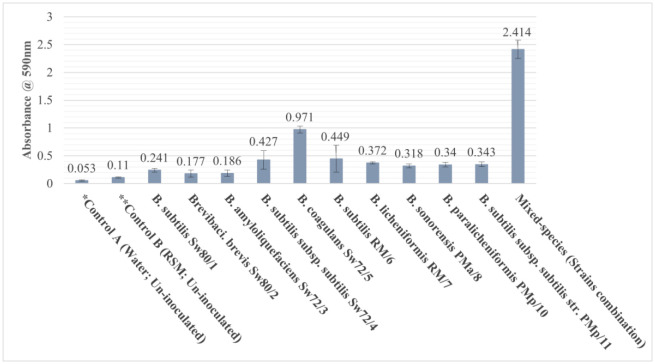
Biofilm formation capabilities of the ten isolates on SS-316 submerged in skim milk. Data represent means ± SE of the obtained results of CV assay from three independent experiments. *Control A is a non-biofilm-containing coupon, incubated in un-inoculated water for background staining determination. **Control B is the fouling layer of milk without bacteria, incubated in un-inoculated skim milk.

Crystal violet (CV) staining assay was used for monitoring the biofilm formation ability of the isolated strains individually and in combination (mixed-species) on SS-316 surfaces in skimmed milk (Figure 1, Table S2). Strains were considered as high, moderate and weak biofilm formers based on the fold value of CV assay in comparison to control B (fouling of milk without bacteria): High biofilm, ≥ 5 fold; Moderate biofilm, 2.5 to 5 fold; Weak biofilm, ≤ 2.5 fold. The results obtained from the biofilm forming assay showed that out of the ten isolates, only *B. coagulans* Sw72/5 exhibited high biofilm formation on the submerged SS surfaces with milk. Sadiq *et al*. [21] reported that the biofilm-forming ability of two strains belonging to *B. coagulans* were higher on the polystyrene microtiter-plate containing TSB than the stainless steel in RSM. However, the strains belonging to *B. subtilis* showed both moderate (strains Sw72/4, RM/6, and PMp/11) and weak (strain Sw80/1) ability to form biofilm on SS-316 coupon. This *Bacillus* species (*B. subtilis*) has previously been characterized as a dairy-associated bacteria reported in milk powders [4],[6],[7], whey WPC80 [15] and sheep milk [30]. Moreover, a moderate ability of biofilm formation was observed by strains *B. licheniformis* RM/7, *B. sonorensis* PMa/8, and *B. paralicheniformis* PMp/10. Previously, Zain *et al*. [15] and Sadiq *et al*. [21] concluded that *B. licheniformis* strains exhibit good biofilm forming ability on SS rather than polystyrene in presence of TSB at 37 °C. Indeed, *B. licheniformis* is the most common contaminant found in dairy-associated environments as well as the final products. Strains *B. sonorensis* and *B. paralicheniformis* are close relatives of *B. licheniformis* and have previously been reported in milk powder [5] and raw milk [30],[31]. The rest of the isolates, *Brevibacillus brevis* Sw80/2 and *Bacillus amyloliquefaciens* Sw72/3, showed weak ability to form biofilm on SS surfaces. Presence of these sporeformer species in milk and dairy products was reported by Sadiq *et al*. [5], Reginensi *et al*. [7] and Vanderkelen *et al*. [29]. Finally, the mixed-species biofilm of the ten isolates was high: 2.4 fold that of the highest single-species alone (strain Sw72/5). The biofilm in natural environments, such as the food industry, consists of a bacterial population (mixed-species biofilms), and it has been found to be more resistant to disinfectants and sanitizers than mono-species biofilms [32]. Thus, according to the observed results, the removal treatments should be conducted on mixed-species biofilms to be closer to the application.

### 3.3. Biosynthesis of metal/metal oxide NPs

The extracellular filtrate of 14 thermoalkali actinobacteria species obtained from their cultivation on the complex broth medium were used as a reducing system for biosynthesis of metal/metal oxide (Ag, CuO and ZnO) NPs from their respective salts. In this process, NPs were produced through a reduction process detected by visual observation as the change in color and precipitation. The primary confirmation of NP biosynthesis was done by the changes in color (Ag, from pale yellow of AgNO_3_ to brown; CuO, from light blue of CuSO_4_ to dark green; ZnO, from colorless ZnSO_4_ to yellowish-white) after addition of the cell free filtrate in equal volumes [33]–[35]. The colors' formation depends on the surface resonance of plasmon. Table 3 shows which reaction mixtures were positive nanoform producers. The produced NPs were 10 different metal/metal oxides, coded according to the metal ion and reducing system (Ag_G310, Ag_G210, Ag_G240, CuO_G215, CuO_G210, CuO_G240, ZnO_G412, ZnO_G710, ZnO_G215 and ZnO_G240).

**Table 3.** Green synthesized metal/metal oxide NPs extracellularly.

Reducing agent			Metal salt solutions		
Strain name	Code	Reference	AgNO_3_ 0.1%	CuSO_4_ 1%	ZnSO_4_ 1%
*Streptomyces* sp. ACD/G413	ACD/G413	[36]			
*Thermoflavimicrobium dichotomicum* HwSW11	X/SW1-1	[37]			
*Laceyella putida* HwSw2	Z/SW2	[38]			
Un-identified thermoalkali actinobacteria	4/G310	[36]	+		
Un-identified thermoalkali actinobacteria	8/G412	[36]			+
*Thermoactinomyces vulgaris* HwFq24	15/FQ24	[38]			
*Streptomyces exfoliates* 15/G710	15/G710	[36]			+
Un-identified thermoalkali actinobacteria	15/S220	[39]			
*Saccharomonospora viridis* Hw G550	20/G550	[20]			
Un-identified thermoalkali actinobacteria	21/G215	[36]		+	+
Un-identified thermoalkali actinobacteria	24/G210	[36]	+	+	
Un-identified thermoalkali actinobacteria	Uk40/G410	[36]			
Un-identified thermoalkali actinobacteria	41/G211	[36]			
Un-identified thermoalkali actinobacteria	215/G240	[36]	+	+	+

### 3.4. Effects of Prot-K and the biosynthesized NPs on dairy biofilms of sporeformers

**Figure 2.**
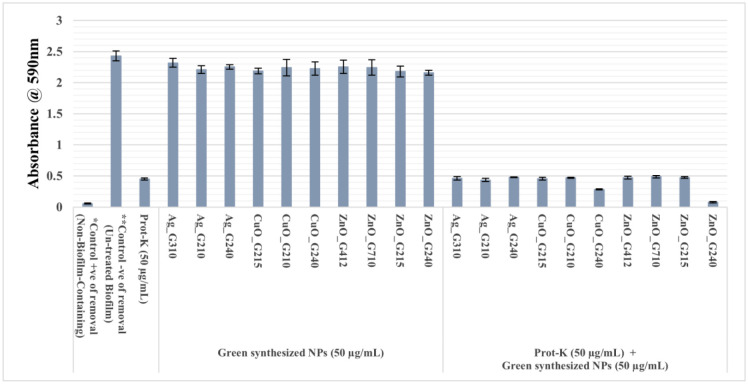
Effects of Prot-K and the biosynthesized NPs on the established biofilms of dairy sporeformers. Values sharing the same letter are not significantly different at *p* < 0.05 using Tukey test. Data represent means ± SE of the obtained results of CV assay from 3 independent experiments. *Control + ve of removal is a non-biofilm-containing coupon, incubated in un-inoculated water instead of RSM to be a reference of maximum removal activity. **Control – ve of removal is a mixed-species biofilm without treatment.

The serine protease Prot-K, typically obtained from *Tritirachium album*, has frequently been used as an efficient biofilm removal agent against those produced by *E. coli*, *G. vaginalis*, *H. influenza*, *L. monocytogenes*, *P. aeruginosa*, *Sal. Gallinarum*, *V. cholerae* and many *Staphylococcus* spp. including MRSA [9],[40],[41]. Activity of metal oxide NPs has also been reported against a range of pathogen-formed biofilms [13]. To our knowledge, this is the first report on the use of Prot-K and biosynthesized NPs for eradication of dairy biofilms of sporeformers. So, in order to evaluate their effects, a model has been established to emulate sporeformers' biofilms in dairy industries: the abovementioned mixed-species biofilm grown on food-grade stainless steel (SS-316). Nguyen and Burrows [42] have reported that the established biofilms on stainless steel can be dispersed by Prot-K at concentrations between 50 and 200 µg/mL. Likewise, the NPs are effective against bacteria in a range of 15 µg/mL to 1400 µg/mL [13]. Accordingly, the treatments of both Prot-K and NPs were carried out at concentrations of 50 µg/mL. Figure 2 and Table S3 show the removal effects of Prot-K and the green synthesized NPs on the established model of dairy spore-type forming biofilms based on absorbance of the de-staining solution of CV assay.

The results showed a significant (P < 0.05) removal effect on the formed biofilm by Prot-K and the combination of Prot-K with ZnO_G240, lettered by “B” and “C”, respectively, according to ANOVA with a Tukey test (Figure 2). However, the synthesized NPs alone did not have effects on the performed biofilms significantly different from the negative control, so they are lettered by “A”. Also, the combinations of Prot-K with the other NPs were non-significant, so they are lettered by “B,” as Prot-K alone. Regarding the NPs CuO_G240, they exhibited removal activity next to ZnO_G240 when combined with Prot-K but share the significance level of Prot-K, so they are lettered by “BC”. Importantly, the observed synergistic effect of Prot-K with ZnO_G240 was interesting and presented the highest significance value: the same as the non-biofilm-containing coupon (control + ve of removal), as both are lettered by “C”. The removal percentages of Prot-K and Prot-K plus the synthesized NPs ZnO_G240, as measured after CV assay and compared to un-treated biofilm (control - ve of removal) and non-biofilm-containing (control + ve of removal) coupons, were 83.76% and 99.19%, respectively (Table S3, Supplementary data).

The known mechanism of biofilm dispersion by Prot-K is cleaving the peptide bonds of aliphatic, aromatic or hydrophobic amino acids in the EPS matrix, which leads to degradation of the protein components and disintegration of the established biofilms [9],[43]. The reactive oxygen species (ROS) is the key mechanism for the action of ZnO NPs against bacterial biofilms. The bacterial contact with ZnO NPs inhibits the respiratory enzyme(s) that facilitate the generation of ROS. The formed ROS can irreversibly damage the bacteria membrane, DNA, mitochondria, etc. [22],[44]. The mechanism of synergism between Prot-K and NPs ZnO_G240 can be inferred from the proteinase K treatment, which significantly degraded the related proteins in the EPS matrix, and the biofilm cells became loose. This enables the ZnO NPs to penetrate deeply inside the biofilm and completely degrade the existing cells. The obtained result of a synergistic effect between Prot-K and NPs (ZnO_G240) is supported by the recent report of Sahli *et al*. [45]. They reviewed the synergistic effect of Prot-K when combined with gold NPs toward biofilm of *P. fluorescens*. Several studies in this line of research have also investigated the synergistic benefits of Prot-K when combined with acylase I [46], antibiotics [11], plant extracts of *R. sativus* [47] and thyme oil [48].

**Figure 3.**
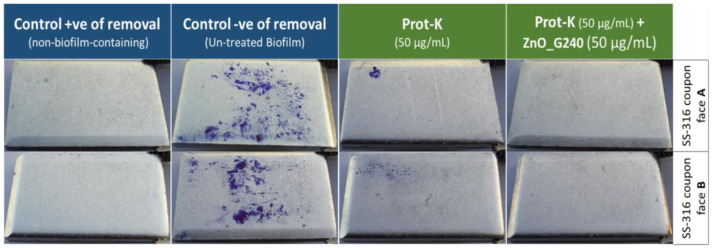
CV staining images for removal of established biofilms by Prot-K and biosynthesized ZnO-NPs. Mixed-species biofilm of ten dairy sporeformers grown on food-grade stainless steel (SS-316) for 72 h were exposed to 50 µg/mL Prot-K alone and in combination with 50 µg/mL of the synthesized NPs ZnO_G240, stained with CV. Control + ve of removal is a non-biofilm-containing coupon, incubated in un-inoculated water instead of RSM to be a reference of maximum removal activity. Control – ve of removal is a mixed-species biofilm without treatment.

It is important to simulate biofilms as formed in dairy plants; thus, the SS-316 coupons (the stainless steel grade that is used widely in dairy production lines) were applied and stained with CV for visualization. Figure 3 displays CV staining images for SS-316 coupons that were captured for both control + ve and – ve of removal and treatment of Prot-K and Prot-K with ZnO_G240. The violet spots of biofilm on SS surfaces of control – ve were decreased markedly with treatment of Prot-K alone, like individual cells or small colonies, and almost disappeared with combination of Prot-K with ZnO_G240. These results confirm the synergistic interaction between Prot-K and the synthesized NPs ZnO_G240, as well as efficiency of the suggested treatment.

### 3.5. Characterization of the effective NPs (ZnO_G240)

**Figure 4.**
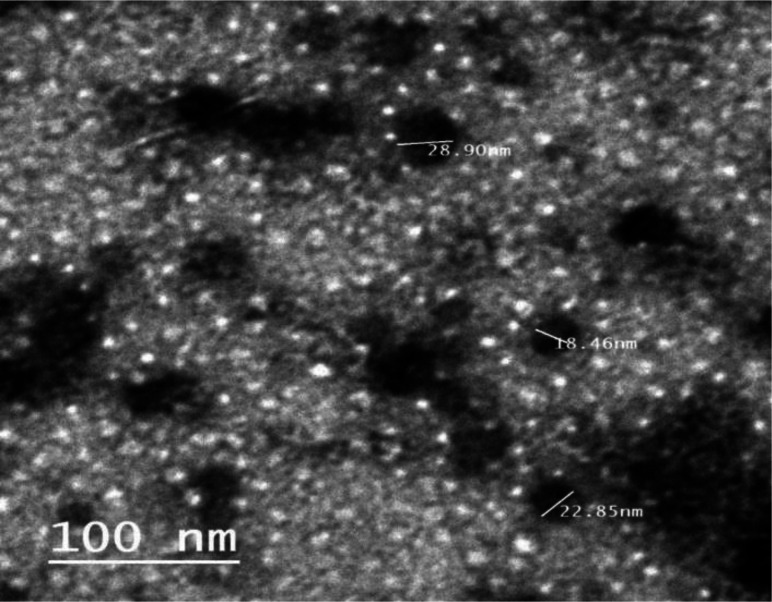
HRTEM micrograph of NPs ZnO_G240. The black areas are the NPs that are ranging between 15 and 29 nm, and the un-measured black areas are NP aggregates.

The biosynthesized NPs ZnO_G240 that exhibit synergism with Prot-K were characterized using High-Resolution Transmission Electron Microscopy (HRTEM), Fourier transform infrared spectroscopy (FTIR) and an X-ray diffraction spectrophotometer (XRD). HRTEM was applied for analysis of the NPs' morphology, size and shape. The image obtained through HRTEM showed that the sizes ranged between 15 and 29 nm, and the NPs had a spherical shape with marginal variation and little aggregation (Figure 4). Similar results have been reported by Vijayakumar *et al*. [49], Al-Shabib *et al*. [50], Ali *et al*. [51], Ishwarya *et al*. [52], for green synthesized ZnO-NPs having anti-biofilm properties.

**Figure 5.**
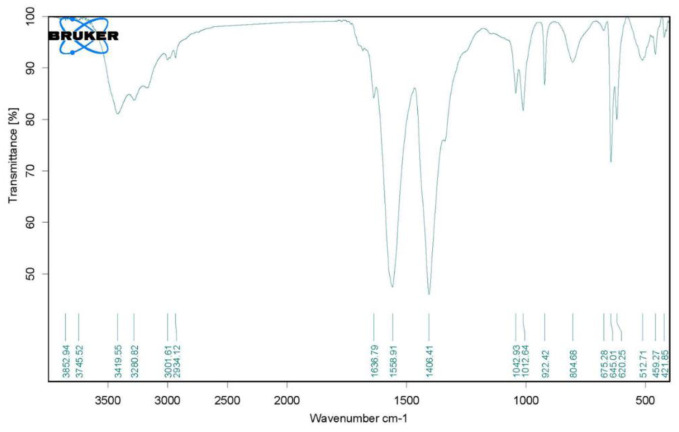
FTIR spectrum of ZnO_G240 nanoparticles.

FTIR measurement was performed to identify the possible biomolecules responsible for reduction, capping and stabilization. The FTIR spectrum of ZnO_G240 NPs shows intense absorption peaks at 3419, 3280, 3001, 2934, 1636, 1558, 1406, 1042, 1012, 922, 804, 675, 645, 620, 512, 459, 421 cm^−1^ (Figure 5). The broad absorptions at 3419 and 3280 cm^−1^ correspond to O-H stretching of the proposed alcohols, flavonoids or phenols present in the extract. The peaks at 3001, 2934 cm^−1^ are C-H stretching of alkenyl and alkyl, respectively, groups of proteins. The absorption bands at 1636 and 1558 cm^−1^ correspond to the amide I and II bands, respectively, that are characteristics of proteins and enzymes. The amide I band is mainly a C=O stretching mode, and the amide II band is a combination of largely N-H in plane bending mode and C-N stretching. The high intensity band at around 1406 cm^−1^ could be attributed to bending vibrations of C-C in aromatic groups of proteins that act here as a protective agent. The important roles of these surrounding proteins observed in FTIR analysis are capping and stabilizing the synthesized nanoparticles. The section between 500 and 900 cm^−1^ is associated with metal oxygen. A similar band pattern has been reported for ZnO-NPs synthesized by green method for biofilm control [49]–[52].

**Figure 6.**
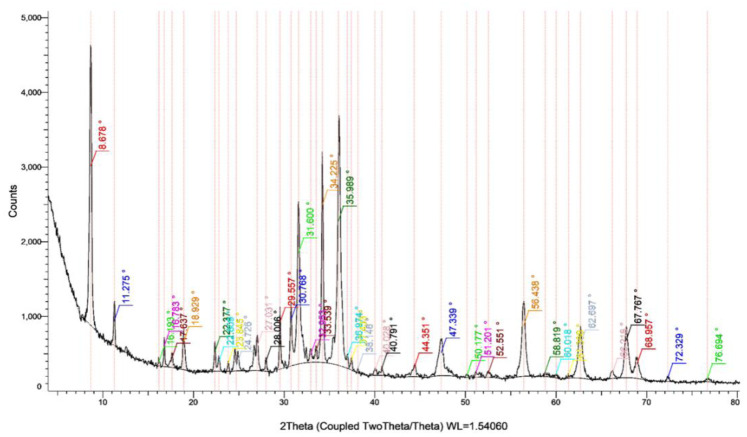
X-ray diffraction pattern of ZnO_G240 nanoparticles.

In the characterization section of XRD analysis of ZnO_G240, the diffractogram showed strong diffraction peaks at 8.6°, 31.6°, 34.2° and 35.9° of 2 theta (Figure 6). XRD spectra indicate that the sample was crystalline with few amorphous phases resulting from proteins and alkanes surrounding the particles as capping agents. The results are in agreement with previous works [53].

### 3.6. Toxicity evaluation of ZnO_G240 NPs by brine shrimp bioassay

**Figure 7.**
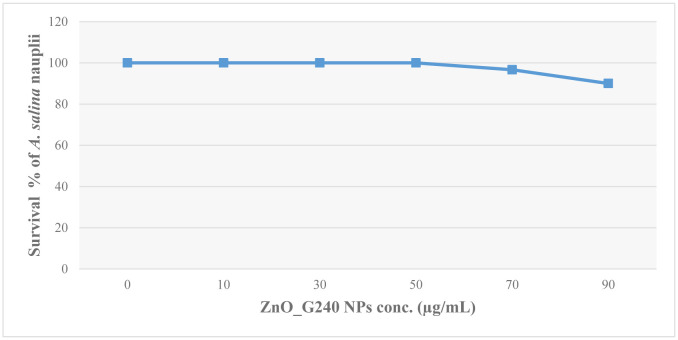
Survival % of *A. salina* (Linnaeus) nauplii after exposure to different conc. of ZnO_G240 NPs.

It is necessary to apply safe and food grade materials in dairy and/or food industry. Thus, in the current experiment, evaluation of the toxicity and biosafety of ZnO-NPs (which are noted as active materials for biofilm eradication) was done. The aquatic organisms brine shrimp were used as bio-monitoring tools in aquatic ecotoxicology, allowing the detection and evaluation of the potential toxicity of actinobacterial ZnO_G240 nanoparticles. The results illustrated in Figure 7 and Table S4 show the ZnO_G240 NPs at the active concentration for biofilm eradication are safe and do not have toxicity. Concerning the effect of high concentration on survival %, it might be caused by light transmission reduction [54].

## 4. Conclusion

In conclusion, the results of this work demonstrated that Prot-K in combination with the biosynthesized NPs ZnO_G240 could be used as a potential cleaning agent for eradication of the dairy biofilms of sporeformers. The current cleaning methods (CIP) in dairy industries are not always sufficient for eradication of the formed biofilms in the production lines, and the proteolytic degradation should be used in conjunction with chemical method for prevention of the re-colonization by the released cells. Thus, the suggested combination could be applied with the CIP regimes to address the dairy biofilm problems.
