# Supplementary material for: A combined treatment of Proteinase K and biosynthesized ZnO-NPs for eradication of dairy biofilm of sporeformers
Source: AIMS Microbiol. 2022 Dec 19;8(4):507–27. doi: 10.3934/microbiol.2022033 (PMC9834087; doi:10.3934/microbiol.2022033)
Supplement: Supplementary file 1 [file microbiol-08-04-033-s001.pdf]

AIMS Microbiology, 8(4): 507–527.
DOI: 10.3934/microbiol.2022033
Received: 11 September 2022
Revised: 24 November 2022
Accepted: 30 November 2022
Published: 19 December 2022

http://www.aimspress.com/journal/microbiology

Research article

A combined treatment of Proteinase K and biosynthesized ZnO-NPs for eradication of dairy biofilm of sporeformers

Ahmed A. Radwan[1], Osama M. Darwesh[2,*], Maha T. Emam[1], Karima A. Mohamed[1] and Hala M. Abu Shady[3]

[1] Genetics and Cytology Dept., National Research Centre (NRC), Cairo, 12622, Egypt
[2] Agricultural Microbiology Dept., National Research Centre (NRC), Cairo, 12622, Egypt
[3] Microbiology Dept., Faculty of Science, Ain-Shams University Cairo, Egypt

* **Correspondence:** Email: darweshosama@yahoo.com, om.darwesh@nrc.sci.eg; Tel: +201155265558.

Supplementary

Table S1. Biochemical profile of the API 50CH/20E kit of the isolated biofilm-forming bacteria.

No.	Biochemical test		Sw80/1	Sw80/2	Sw72/3	Sw72/4	Sw72/5	RM/6	RM/7	PMa/8	PMp/10	PMp/11
1	GLY	Glycerol	+	+	+	+	+	+	+	+	+	+
2	ERY	Erythritol	-	-	-	-	-	-	-	-	-	-
3	DARA	D-Arabinose	-	-	-	-	-	-	-	-	-	-
4	LARA	L-Arabinose	+	+	+	+	+	+	+	+	+	+
5	RIB	D-Ribose	+	+	+	+	+	+	+	+	+	+
6	DXYL	D-Xylose	+	+	+	+	+	+	+	+	+	+
7	LXYL	L-Xylose	-	-	-	-	-	-	+	-	-	-
8	ADO	D-Adonitol	-	-	-	-	-	-	-	-	-	-
9	MDX	Methyl-Beta-D-Xylopyranoside	-	-	-	-	-	-	-	-	-	-
10	GAL	D-Galactose	-	-	-	-	+	-	+	+	+	+
11	GLU	D-Glucose	+	+	+	+	+	+	+	+	+	+
12	FRU	D-Fructose	+	+	+	+	+	+	+	+	+	+
13	MNE	D-Mannose	+	+	+	+	+	+	+	+	+	+
14	SBE	L-Sorbose	-	-	-	-	-	-	+	+	-	-
15	RHA	L-Rhamnose	-	-	-	-	-	-	-	-	-	-
16	DUL	Dulcitol	-	-	-	-	-	-	-	-	-	-
17	INO	Inositol	+	-	+	-	-	+	+	+	+	+
18	MAN	D-Mannitol	+	+	+	+	+	+	+	+	+	+
19	SOR	D-Sorbitol	+	+	+	+	+	+	+	+	+	+
20	MDM	Methyl-Alpha-D-Mannopyranoside	-	-	-	-	-	-	-	-	-	-
21	MDG	Methyl-Alpha-D-Glucopyranoside	+	+	+	+	+	+	+	+	+	+
22	NAG	N-Acetylglucosamine	+	-	-	+	+	-	+	+	+	-
23	AMY	Amygdalin	+	+	+	+	+	+	+	+	+	+
24	ARB	Arbutin	+	+	+	+	+	+	+	+	+	+
25	ESC	Esculin Ferric Citrate	+	+	+	+	+	+	+	+	+	+
26	SAL	Salicin	+	+	+	+	+	+	+	+	+	+
27	CEL	D-Cellobiose	+	+	+	+	+	+	+	+	+	+
28	MAL	D-Maltose	+	+	+	+	+	+	+	+	+	+
29	LAC	D-Lactose (Bovine Origin)	-	-	-	-	+	-	-	-	-	-
30	MEL	D-Melibiose	-	-	-	-	+	-	-	-	-	-
31	SAC	D-Saccharose (Sucrose)	+	+	+	+	+	+	+	+	+	+
32	TRE	D-Trehalose	+	+	+	+	+	+	+	+	+	+
33	INU	Inulin	+	+	+	+	+	+	-	-	+	+
34	MLZ	D-Melezitose	-	-	-	-	-	-	-	-	-	-
35	RAF	D-Raffinose	-	-	-	-	+	-	-	-	-	-
36	AMD	Amidon (Starch)	+	+	+	+	+	+	-	-	+	+
37	GLYG	Glycogen	+	+	+	+	+	+	-	-	+	+
38	XLT	Xylitol	-	-	-	-	-	-	-	-	-	-
39	GEN	Gentiobiose	+	+	+	+	+	+	-	-	+	+
40	TUR	D-Turanose	-	-	-	-	+	-	-	+	+	-
41	LYX	D-Lyxose	-	-	-	-	-	-	-	-	-	-
42	TAG	D-Tagatose	-	-	-	-	-	-	+	+	+	+
43	DFUC	D-Fucose	-	-	-	-	-	-	-	-	-	-
44	LFUC	L-Fucose	-	-	-	-	-	-	-	-	-	-
45	DARL	D-Arabitol	-	-	-	-	+	-	-	-	-	-
46	LARL	L-Arabitol	-	-	-	-	-	-	-	+	-	-
47	GNT	Potassium Gluconate	-	-	-	-	+	-	-	+	-	+
48	2KG	Potassium 2-Ketogluconate	-	-	-	-	-	-	-	-	-	-
49	5KG	Potassium 5-Ketogluconate	-	-	-	-	-	-	+	+	-	-
50	ONPG	B-Galactosidase	+	+	+	+	+	+	+	+	+	+
51	ADH	Arginine Dihydrolase	-	-	-	-	-	-	+	+	+	-
52	LDC	Lysine Decarboxylase	-	-	-	-	-	-	-	-	-	-

Continued on next page

No.	Biochemical test		Sw80/1	Sw80/2	Sw72/3	Sw72/4	Sw72/5	RM/6	RM/7	PMa/8	PMp/10	PMp/11
53	ODC	Ornithine Decarboxylase	-	-	-	-	-	-	-	-	-	-
54	CIT	Citrate Utilization	-	+	-	+	+	-	-	-	+	-
55	H2S	Hydrogen Sulfide Production	-	-	-	-	-	-	-	-	-	-
56	URE	Urease	-	+	-	-	-	-	-	-	-	-
57	TDA	Tryptophan Deaminase	-	-	-	-	-	-	-	-	-	-
58	IND	Indole Production	-	-	-	-	-	-	-	-	-	-
59	VP	Voges-Proskauer	-	-	-	+	-	-	+	+	+	+
60	GEL	Gelatinase	+	+	+	+	+	+	+	+	+	+
61	NIT	Nitrate Reduction	+	+	+	-	+	+	-	+	-	-

Table S2. Biofilms formation capability of the ten isolates on SS-316 submerged in milk.

	Absorbance @ 590nm			Mean	Std. Error	***Biofilm-formation %	Fold (comparing to control B)
*Control A (Water; Un-inoculated)	0.041	0.063	0.055	0.053	0.0111	0	0.48
**Control B (RSM media; Un-inoculated)	0.107	0.119	0.104	0.11	0.00794	107.55	1
B. subtilis str. Sw80/1	0.223	0.22	0.28	0.241	0.0338	354.72	2.19
Brevibaci. brevis str. Sw80/2	0.25	0.151	0.13	0.177	0.0641	233.96	1.61
B. amyloliquefaciens str. Sw72/3	0.251	0.152	0.156	0.186	0.056	250.94	1.70
B. subtilis subsp. *subtilis* str. Sw72/4	0.325	0.623	0.333	0.427	0.17	705.66	3.88
B. coagulans str. Sw72/5	0.903	0.986	1.024	0.971	0.0619	1732.08	8.83
B. subtilis str. RM/6	0.728	0.329	0.29	0.449	0.242	747.17	4.08
B. licheniformis str. RM/7	0.393	0.35	0.374	0.372	0.0215	601.89	3.38
B. sonorensis str. PMa/8	0.332	0.277	0.344	0.318	0.0357	500	2.89
B. paralicheniformis str. PMp/10	0.367	0.359	0.293	0.34	0.0406	541.51	3.09
B. subtilis subsp. *subtilis* str. PMp/11	0.311	0.327	0.391	0.343	0.0423	547.17	3.12
Mixed-species (Strains combination)	2.402	2.256	2.584	2.414	0.164	4454.72	21.95

*Note: *Control A is a non-biofilm-containing coupon, SS-316 coupon incubated in un-inoculated water for background staining determination. **Control B is the fouling layer of milk without bacteria, SS-316 coupon incubated in un-inoculated skim milk. ***The percentages of biofilm-formation capacity were calculated using the formula [The mean absorbance of (test - control A)/control A] ×100.

Table S3. Effect of Prot-K and the biosynthesized NPs on the established biofilms of dairy sporeformers.

		Absorbance @ 590nm			Mean	Std. Error	Removal %
Control + ve of removal (Non-Biofilm-Containing)		0.066	0.042	0.061	0.0563	0.00731	100
Control - ve of removal (Un-treated Biofilm)		2.559	2.448	2.283	2.43	0.0802	2.32
Prot-K (50 µg/mL)		0.483	0.43	0.441	0.451	0.0161	83.76
Green synthesized NPs (50 µg/mL)	Ag_G310	2.186	2.424	2.348	2.319	0.0702	6.88
	Ag_G210	2.093	2.248	2.289	2.21	0.0597	11.37
	Ag_G240	2.196	2.324	2.242	2.254	0.0374	9.56
	CuO_G215	2.223	2.101	2.238	2.187	0.0434	12.32
	CuO_G210	2.068	2.506	2.152	2.242	0.134	10.05
	CuO_G240	2.028	2.405	2.244	2.226	0.109	10.71
	ZnO_G412	2.361	2.366	2.042	2.256	0.107	9.48
	ZnO_G710	2.21	2.044	2.478	2.244	0.126	9.97
	ZnO_G215	2.328	2.025	2.186	2.18	0.0875	12.60
	ZnO_G240	2.083	2.191	2.208	2.161	0.0391	13.39
Prot-K (50 µg/mL) + Green synthesized NPs (50 µg/mL)	Ag_G310	0.403	0.505	0.475	0.461	0.0303	83.34
	Ag_G210	0.412	0.404	0.485	0.434	0.0258	84.46
	Ag_G240	0.486	0.464	0.482	0.477	0.00677	82.69
	CuO_G215	0.426	0.504	0.439	0.456	0.0241	83.55
	CuO_G210	0.481	0.474	0.455	0.47	0.00777	82.98
	CuO_G240	0.286	0.293	0.269	0.283	0.00713	90.67
	ZnO_G412	0.428	0.478	0.512	0.473	0.0244	82.85
	ZnO_G710	0.506	0.448	0.509	0.488	0.0199	82.23
	ZnO_G215	0.449	0.479	0.494	0.474	0.0132	82.81
	ZnO_G240	0.061	0.069	0.098	0.076	0.0112	99.19

*Note: The removal percentage was calculated using the formula [The mean absorbance of ((control negative - test) + control positive)/ control negative] ×100, where control negative is a mixed-species biofilm without treatment and control positive is a non-biofilm-containing coupon, incubated in un-inoculated water instead of RSM to be a reference of maximum removal activity.

Table S4. Toxicity assay of the synthesized ZnO_G240 NPs by *A. salina* (Linnaeus) nauplii.

ZnO_G240 NPs conc. (µg/mL)	Number of surviving nauplii			Mean	Std. Error	Survival % of *A. salina* (Linnaeus) nauplii
0	10	10	10	10	0	100
10	10	10	10	10	0	100
30	10	10	10	10	0	100
50	10	10	10	10	0	100
70	10	9	10	9.667	0.577	96.67
90	9	9	9	9	0	90

*Note: The survival percentage was calculated using the formula [Number of nauplii at (Teast/negative control (NPs conc. 0 µg/mL))] ×100.

AIMS Press

© 2022 the Author(s), licensee AIMS Press. This is an open access article distributed under the terms of the Creative Commons Attribution License (http://creativecommons.org/licenses/by/4.0)
